# NANOG expression in parthenogenetic porcine blastocysts is required for intact lineage specification and pluripotency

**DOI:** 10.5713/ab.23.0210

**Published:** 2023-08-28

**Authors:** Mingyun Lee, Jong-Nam Oh, Gyung Cheol Choe, Kwang-Hwan Choi, Dong-Kyung Lee, Seung-Hun Kim, Jinsol Jeong, Yelim Ahn, Chang-Kyu Lee

**Affiliations:** 1 Department of Agricultural Biotechnology, Animal Biotechnology Major, and Research Institute of Agriculture and Life Sciences, Seoul National University, Seoul 08826, Korea; 2 Department of Cellular and Molecular Physiology, Yale School of Medicine, New Haven, CT 06510, USA; 4 Institute of Green Bio Science and Technology, Seoul National University, Pyeongchang 25354, Korea

**Keywords:** Embryo, Lineage Specification, NANOG, Pig, Pluripotency, Proliferation

## Abstract

**Objective:**

Nanog homeobox (NANOG) is a core transcription factor that contributes to pluripotency along with octamer binding transcription factor-4 (OCT4) and sex determining region-Y box-2 (SOX2). It is an epiblast lineage marker in mammalian pre-implantation embryos and exhibits a species-specific expression pattern. Therefore, it is important to understand the lineage of NANOG, the trophectoderm, and the primitive endoderm in the pig embryo.

**Methods:**

A loss- and gain-of-function analysis was done to determine the role of NANOG in lineage specification in parthenogenetic porcine blastocysts. We analyzed the relationship between NANOG and pluripotent core transcription factors and other lineage makers.

**Results:**

In *NANOG*-null late blastocysts, OCT4-, SOX2-, and SOX17-positive cells were decreased, whereas GATA binding protein 6 (GATA6)-positive cells were increased. Quantitative real-time polymerase chain reaction revealed that the expression of SOX2 was decreased in *NANOG*-null blastocysts, whereas that of primitive endoderm makers, except SOX17, was increased. In NANOG-overexpressing blastocysts, caudal type homeobox 2 (CDX2-), SOX17-, and GATA6-positive cells were decreased. The results indicated that the expression of primitive endoderm markers and trophectoderm-related genes was decreased.

**Conclusion:**

Taken together, the results demonstrate that NANOG is involved in the epiblast and primitive endoderm differentiation and is essential for maintaining pluripotency within the epiblast.

## INTRODUCTION

Nanog homeobox (NANOG) is a core transcription factor associated with the pluripotent state and plays an important role in maintaining pluripotency in mouse and human embryonic stem cells (ESCs) [1–4]. Several studies have revealed a role for NANOGs in the pluripotency of mammalian ESCs. For example, overexpression of NANOG enables mouse ESCs to maintain pluripotency under feeder- and LIF-free conditions [5]. Moreover, the proximal *Nanog* promoter is activated by the octamer binding transcription factor-4 – sex determining region-Y box-2 (OCT4-SOX2) complex to control pluripotency and cell differentiation [6]. There are also studies indicating that NANOG and caudal type homeobox 2 (CDX2) bind to and suppress the promoters of one another [7,8]. NANOG also plays a role in regulating S-phase entry in human ESCs by binding to cyclin dependent kinase 6 (*CDK6*) and cell division cycle 25A (*CDC25A*) genes [9]. Therefore, NANOG plays an important role in the maintenance of pluripotency and lineage specification in mammalian ESCs.

There are two lineage specifications for embryogenesis during mammalian pre-implantation [10]. The first is divided into the inner cell mass (ICM) and the trophectoderm (TE), whereas the second is divided into the epiblast and primitive endoderm. NANOG is an epiblast marker associated with the second lineage specification [11]. In *Nanog*-deficient mouse embryos, it was revealed that ICM did not differentiate into epiblasts and only differentiated into parietal endoderm-like cells [2]. In subsequent study, intact primitive endoderms are not formed in *Nanog*-null mouse embryos [12]. In bovine embryos, NANOG was required for epiblast formation and the maintenance of pluripotency [13]. Because NANOG has important functions, it is conserved in rodents and primates [14], however, other characteristics of NANOG in pre-implantation embryos are species-specific. In the case of pigs [15], the expression of NANOG begins in the blastocyst, which is divided into the ICM and TE, whereas it occurs in the full blastocyst in humans [16], in the eight-cell stage in cattle [17] in the morula stage in rabbit [18], and in the two-cell stage in mice [19]. Another study indicated that a significant fraction of the binding sites for OCT4 and NANOG in mouse and human ESCs is not conserved [20]. Furthermore, NANOG is required for primitive endoderm formation in mouse embryos [12], but primitive endoderm formation does not depend on NANOG in bovine embryos [21]. Other pluripotent-related traits differ from species to species [22–24]; therefore, the species-specific characteristics of NANOG should be evaluated in each animal. Pigs are considered a model animal because of their anatomical and physiological similarity to humans [25,26]. Moreover, among the animals mentioned above, NANOG expression in pigs and humans in pre-implantation embryos is similar. So, in this study, the role of NANOG during parthenogenetic porcine blastocyst formation was determined. First, we analyzed the expression profiles and relationships within the ICM of NANOG, GATA binding protein 6 (GATA6), and SOX17. We then used the clustered regularly interspaced short palindromic repeats/CRISPR-associated 9 (CRISPR/Cas9) system and analyzed the characteristics of the NANOG-targeted blastocyst by immunostaining and quantitative polymerase chain reaction (qPCR). Finally, the expression patterns of other genes were evaluated by overexpressing NANOG at the eight-cell stage using lipofectamine. Our findings provide insight into the role of NANOG in lineage specification and pluripotency in pre-implantation porcine embryos.

## MATERIALS AND METHODS

The care and experimental use of pigs were approved by the Institute of Laboratory Animal Resources, Seoul National University (SNU-140328-2). Unless otherwise stated, all chemicals were obtained from Sigma–Aldrich Corp. (St. Louis, MO, USA).

### *In vitro* embryo production

The ovaries from prepubertal gilts were obtained from a local slaughterhouse (Anyang, Korea) and transferred to the laboratory in warm saline. Cumulus-oocyte complexes (COCs) were collected by aspirating 3 to 7 mm follicles of prepubertal gilts using a 10-mL syringe containing an 18-gauge needle. Sediments were washed with TL–HEPES–PVA medium and oocytes with compact cumulus cells and a granulated cytoplasm were selected for *in vitro* maturation. The washed COCs were cultured in tissue culture medium (TCM-199; Life Technologies, Carlsbad, CA, USA) containing 10 ng/mL of epidermal growth factor, 1 mg/mL of insulin, and 10% porcine follicular fluid for 44 h at 39°C in 5% CO_2_ and 100% humidity. The COCs were matured with 10 IU/mL of gonadotropin hormone, pregnant mare serum gonadotropin (Lee Biosolutions, Maryland Heights, MO, USA), and human chorionic gonadotropin for the first 22 h. The COCs were then matured under hormone-free conditions. To generate parthenotes, cumulus-free oocytes were activated with an electric pulse (1.0 kV/cm for 60 ms) in inactivation medium (280 mM mannitol, 0.01 mM CaCl_2_, 0.05 mM MgCl_2_) using a BTX Electrocell Manipulator (BTX, CA, USA), followed by 4 h of incubation in PZM3 medium containing 2 mM 6-dimethylaminopurine.

### Production of CRISPR/Cas9 vectors and *NANOG* mRNA

The candidate targeting sequence against the pig *NANOG* gene was selected using a CRISPR gRNA design tool (https://chopchop.cbu.uib.no) to improve gene-targeting efficiency and minimize off-targeting effects. DNA oligonucleotides carrying the target sequences were constructed by adding PAM sequences (Supplementary Table S1). The candidate DNA construct for NANOG was inserted into the pX330 plasmid and validated using the pCAG-EGxxFP reporter system [27]. The NANOG-pX330 and pCAG-EG(pig NANOG)FP constructs were introduced into porcine fetal fibroblast (pFF) cells in 12-well plates (300 ng/well) using lipofectamine 3000 reagent (Thermo Fisher Scientific, Waltham, MA, USA). Enhanced green fluorescent protein (EGFP) fluorescence was observed under a fluorescence microscope 48 h after transfection. A thermal cycler was used for target sequencing of the genomic DNA of single microinjected blastocysts. After destroying cells by repeated high and low temperatures in ultrapure water, PCR was performed using porcine NANOG gDNA-specific primers (Supplementary Table S2) and 2× PCR master mix solution (iNtRON Biotechnology, Seongnam, Korea). The amplified PCR products were analyzed using an ABI PRISM 3730 DNA Analyzer (Applied Biosystems, Foster, CA, USA). Finally, the selected guide sequence (sgRNA) was synthesized using a CRISPR/Cas9 service (Cosmo Genetech, Seoul, Korea).

Total porcine RNA was extracted from porcine ESCs using TRIzol1 reagent (Invitrogen, San Diego, CA, USA) according to the manufacturer’s instructions. Complementary DNA was synthesized using a High-Capacity RNA-to cDNA Kit (Applied Biosystems, USA) according to the manufacturer’s instructions in a final volume of 20 μL. Porcine NANOG cDNA was cloned using 2× PCR master mix solution (iNtRON Biotechnology, Korea) and porcine NANOG cDNA-specific primers. Amplified PCR products were TA-cloned and analyzed using an ABI PRISM 3730 DNA Analyzer (Applied Biosystems, USA). Porcine NANOG mRNA was synthesized and modified (RNA coding sequence, 5′ cap, 3′poly A tail, and 5′ & 3′ UTR) by Bioneer (Daejeon, Korea).

### Microinjection of RNA into parthenotes

For the pNANOG CRISPR/Cas9 knockout assay, 1 μL of 20 ng/μL commercial Cas9 mRNA (Thermo Fisher Scientific, USA) and 1 μL of 10 ng/μL sgRNA were added to 8 μL of Media-199 (Gibco, Waltham, MA, USA). One hour after parthenogenetic activation, the embryos at the one-cell stage were injected with 2 pL of RNA solution in manipulation media. The microinjection procedure was conducted using a micromanipulator (Eclipse TE2000; Nikon Tokyo, Japan) with a Femtotip II microinjector (Eppendorf, Hamburg, Germany). After microinjection, the embryos were washed and cultured in PZM3 media for 6 days.

### Lipofection of mRNA into porcine eight-cell stage embryos using zona removal

For pNANOG overexpression at the D5 blastocyst formation stage, 20 ng of pNANOG mRNA, opti MEM (Gibco, USA), and MessengerMAX reagent mRNA (Thermo Fisher Scientific, USA) was brought to 20 μL with PZM3 media at the morula stage.

### Immunocytochemistry

Each embryo stage without the zona pellucida was fixed in 4% paraformaldehyde for 15 min at room temperature. The fixed samples were permeabilized using 1% Triton X-100 for 1 h at room temperature and washed three times with phosphate-buffered saline (PBS). The embryos were blocked using 10% goat or donkey serum in PBS for 1 h at room temperature. The samples were stained with anti-SOX2 (5 μg/mL), anti-NANOG (1 μg/mL), anti-OCT4 (1 μg/mL), anti-SOX17 (1 μg/mL), anti-CDX2 (1 μg/mL), or anti-GATA6 (1 μg/mL) antibodies in PBS containing 10% goat serum or donkey serum at 4°C overnight (Supplementary Table S3). After washing three times in washing solution (PBS containing 0.2% Tween-20 and 1% bovine serum albumin for 10 min), the embryos were incubated with goat anti-rabbit Alexa 488 (Invitrogen, USA), anti-rabbit Alexa 555 (Invitrogen, USA), or donkey anti-rabbit Alexa 647 (Invitrogen, USA) antibodies in PBS containing 10% goat or donkey serum at RT for 1 h. The samples were washed three times with washing solution after secondary antibody treatment. For immunostaining with the three antigens together, three primary antibodies were applied to the samples individually. The immunostained embryos were mounted onto a glass slide with Prolong Gold containing 4′,6-diamidino-2-phenylindole (DAPI) (Invitrogen, USA) and cured for more than 24 h. Supplementary Table S3 lists the antibodies used. Images of the stained cells were captured using an inverted fluorescence microscope and processed by the ImageJ program. ImageJ was used to merge the fluorescence images and to measure the fluorescence staining intensity.

### Confocal imaging process

**Figure 1:**
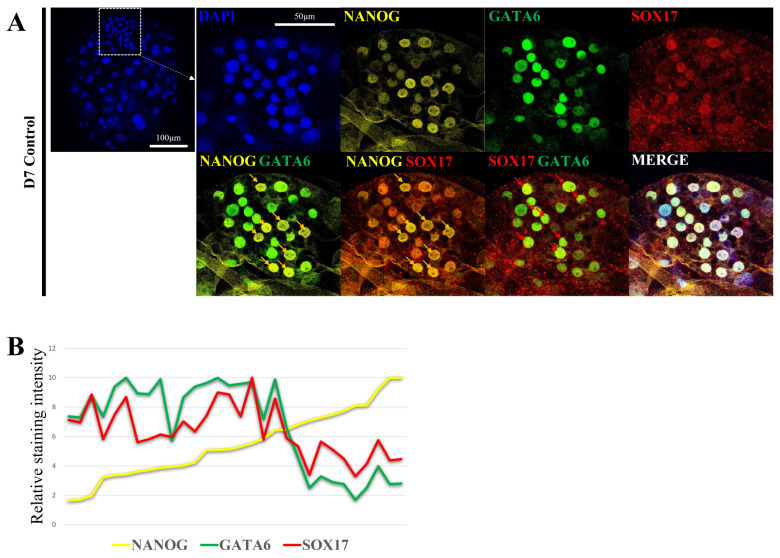
Lineage marker expression pattern in porcine D7 blastocyst stage. (A) Expression and localization of lineage marker genes (*NANOG*, *GATA6*, and *SOX17*) in porcine D7 blastocysts. Nuclei were stained with DAPI using yellow for *NANOG*, green for *GATA6*, and red for *SOX17*. The size marker corresponds to 100 μm. (B) Relative staining intensity for *NANOG*, *GATA6*, and *SOX17* in Porcine D7 blastocysts ICM. *NANOG*, Nanog homeobox; *GATA6*, GATA binding protein 6; *SOX17*, sex determining region-Y box-2; DAPI, 4′, 6-diamidino-2-phenylindole; ICM, inner cell mass.

Confocal immunofluorescence images were captured with a Leica SP8X (Leica Microsystem, Wetzlar, Germany) and processed with LAS X software, and the relative fluorescence intensity was quantified using ImageJ software [28,29]. In Figure 1B, the staining intensity of NANOG was sorted in the order of the lowest cell, and the relative staining intensity was calculated by setting the highest staining intensity to 10 for all three genes. Staining intensity was measured in 30 cells included in ICM in one blastocyst to determine the exact relationship.

### Quantitative real-time polymerase chain reaction

Total RNA from pooled embryos at each stage of *in vitro*-produced embryos (blastocysts, n = 10) was isolated using an Arcturus PicoPure RNA Isolation Kit (Applied Biosystems, USA) according to the manufacturer’s instructions. Complementary DNA (cDNA) was synthesized using a High-Capacity RNA-to-cDNA Kit (Applied Biosystems, USA). The cDNA samples were amplified using Power SYBR Green Master Mix (Applied Biosystems, USA) containing 1 pmol of each primer set listed in Supplementary Table S2 in a 10 μL reaction volume. Amplification and detection were conducted using the ABI 7300 Real-Time PCR System (Applied Biosystems, USA) under the following conditions: one cycle at 50°C for 2 min and 95°C for 10 min, followed by 40 cycles of denaturation at 95°C for 15 s and annealing/extension for 1 min (annealing/extension temperatures were dependent on each primer set). The dissociation curves were analyzed and the amplified products were analyzed by agarose gel electrophoresis to confirm the size of the PCR products. The relative expression levels were calculated by normalizing the threshold cycle (Ct) values of each gene to that of the reference gene beta-actin (ACTB) using the delta-delta Ct method.

### Statistical analysis

Statistical analysis of the data was performed using GraphPad Prism Software (version 7; San Diego, CA, USA). Significant differences in gene expression among the experimental groups were determined by a one-way analysis of variance followed by Tukey’s multiple comparison test. Differences were considered significant at p<0.05 (* p<0.05 and ** p<0.01, *** p<0.001, **** p<0.0001 in the Figures). Data are presented as the mean±standard error of the mean.

## RESULTS

### Lineage marker expression patterns in porcine blastocysts

Second lineage specification and the role of NANOG in porcine pre-implantation embryogenesis are not well defined. Therefore, the epiblast and primitive endoderm were analyzed by confocal microscopy to determine the location and level of expression of each marker gene (Figure 1A). Embryo production by parthenogenetic activation were used to reduce sperm-derived variables. With respect to the D7 blastocyst ICM, some cells exhibited high NANOG fluorescence intensity (yellow arrow), whereas other had low NANOG fluorescence intensity. GATA6 and SOX17 are primitive endoderm markers. They were expressed at a low level in cells with high NANOG fluorescence intensity and at a high level in cells with low NANOG fluorescence intensity (red arrow). Based on these results, ICM may be divided into two groups: a group with high NANOG fluorescence intensity and a group with high SOX17 and GATA6 fluorescence intensity (Figure 1B).

### Effects of CRISPR/Cas9 targeting NANOG on embryo development

**Figure 2:**
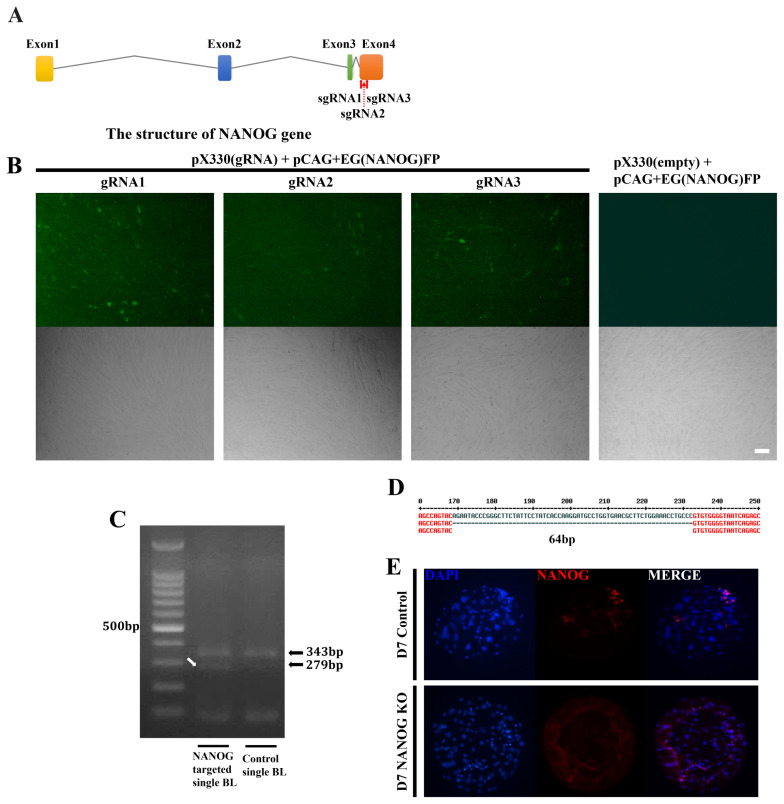
gRNA validation for NANOG editing using the CRISPR/Cas9 system. (A) Schematic representation of the porcine NANOG locus and the CRISPR/Cas9 targeting sequences of gRNA1 and gRNA3. (B) The cleavage efficiency of pX330 with gRNAs 1 to 3 in porcine fetal fibroblasts. Co-transfection of the pCAG-EGxxFP vector containing a portion of the porcine NANOG CDS and pX330 vectors. The size marker corresponds to 50 μm. (C) Agarose gel electrophoresis of the PCR products of genomic DNA from single NANOG-targeted D7 blastocysts. The white arrow indicates the shortened NANOG genomic DNA from the dual targeting system. (D) Sanger sequencing results of the white arrow DNA band in Figure 2C. (E) Immunofluorescence analysis for NANOG (red) and DAPI nuclear staining in NANOG-targeted D7 blastocysts. The size marker corresponds to 100 μm. *NANOG*, Nanog homeobox; CRISPR/Cas9, clustered regularly interspaced short palindromic repeats/CRISPR-associated 9; CDS, coding sequence; PCR, polymerase chain reaction; DAPI, 4′, 6-diamidino-2-phenylindole.

The CRISPR/Cas9 system was used to analyze the role of NANOG in this lineage specification at the D7 blastocyst stage. Candidate sequences were selected that were expected to have the highest efficiency among the four NANOG exons (Figure 2A). Of the selected target sequences, the most effective, gRNA1 and gRNA3, were selected using the EGxxFP system in porcine fetal fibroblasts (Figure 2B). After injection of Cas9 mRNA and sgRNA into the one-cell stage embryo, the genomic DNA of the D7 blastocyst was purified and the target sequence was amplified by PCR (Figure 2C). As a result, a DNA band with a sequence shorter than that of the control single blastocyst was observed. Sanger sequencing of this DNA band confirmed that a 64 bp fragment was deleted between the two target sequences (Figure 2D). NANOG knockout was confirmed as there were no NANOG-positive cells based on immunostaining of the D7 blastocysts (Figure 2E). When the knockout efficiency was confirmed by immunofluorescence in NANOG targeted blastocysts, NANOG expression was not detected in 65.9% of blastocysts (56 out of 85 blastocysts). There was no significant difference in embryo cleavage rate and blastocyst formation rate in the NANOG targeted embryos compared to the control group in which only Cas9 mRNA was injected (Supplementary Table S4).

**Figure 3:**
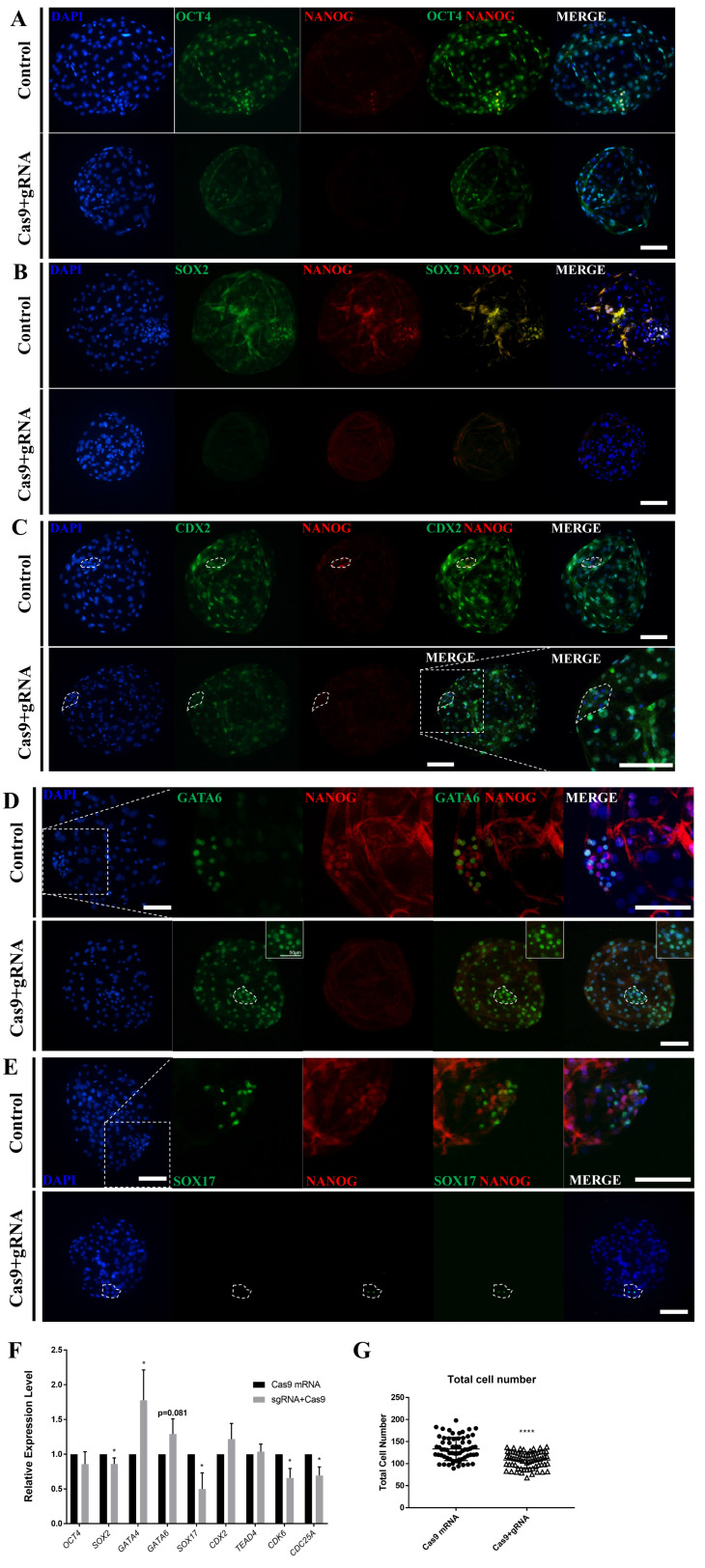
Effects of NANOG knockout on lineage marker genes in porcine blastocysts. (A)–(E) Immunofluorescence analysis for DAPI and NANOG (red), as well as OCT4, SOX2, CDX2, GATA6, and SOX17 (green) in control and NANOG-targeted porcine D7 blastocysts. The white dotted ellipse indicates the ICM. The size marker corresponds to 100 μm. The sample size was *n* = 10 for each group. (F) Transcription levels of epiblast, primitive endoderm, and trophectoderm-related genes shown for control and NANOG-targeted D7 blastocysts. The sample size was *n* = 30. Each group contained three replicates. Error bars represent the mean standard error of the mean. * Corresponds to significant differences (* p<0.05). (G) The number of the total cells in NANOG targeted blastocysts. **** Corresponds to significant differences (**** p<0.0001). NANOG, Nanog homeobox; DAPI, 4′, 6-diamidino-2-phenylindole; OCT4, octamer binding transcription factor-4; SOX2, sex determining region-Y box-2; CDX2, caudal type homeobox 2; GATA6, GATA binding protein 6.

To determine the role of NANOG in the late-stage porcine blastocyst, an immunofluorescence analysis was performed. First, ICM formation was confirmed in the NANOG knockout D7 blastocysts by dual staining for OCT4 and NANOG (Figure 3A); however, NANOG knockout decreased the staining intensity of OCT4 and decreased the number of SOX2-positive cells (Figure 3A, 3B), (Table 1). CDX2, a trophectoderm marker, was also observed in the ICM-like clusters of NANOG-targeted blastocysts (Figure 3C). Compared with the control group, the NANOG-targeted group generally expressed GATA6 in the ICM and also expressed in the trophectoderm (Figure 3D). Contrary to expectations, SOX17 exhibited limited expression in some ICM cells of the NANOG-targeted blastocyst (Figure 3E). Quantitative PCR analysis revealed that there was no significant difference in the expression of *OCT4* in the NANOG-targeted blastocyst; however, the expression of *SOX2* was decreased (Figure 3F). The expression of *GATA4* and *GATA6* was significantly increased, whereas that of *SOX17* was decreased. Trophectoderm-related genes were unaffected. CDK6 and CDC25A are known to be involved in S-phase regulation by direct binding with NANOG [9], and their expression was decreased in the NANOG targeted group. Based on the above results, the NANOG-targeted blastocyst is capable of ICM formation; however, the normal epiblast lineage was disturbed. Conversely, a portion of the primitive endoderm-like cells and the expression of some lineage markers were increased. The NANOG targeted embryo group significantly reduced the total cell number of blastocysts compared to the control group, suggesting that NANOG affects embryonic cell proliferation.

### Effects of NANOG overexpression on embryo development

We injected porcine NANOG mRNA at the one-cell stage into porcine embryos to evaluate the role of NANOG using an overexpression assay. Compared with the control group, the group injected with NANOG mRNA showed a significantly lower blastocyst formation rate (Supplementary Figure S1A). NANOG mRNA expression was lower in the overexpression group than in the control group (Supplementary Figure S1B). Therefore, we designed an overexpression assay to match the normal NANOG expression time and the artificial overexpression time. The zona pellucida of eight-cell stage embryos was removed and porcine NANOG mRNA was overexpressed using lipofectamine (Supplementary Figure S1C). Using EGFP mRNA, blastocysts with a zona pellucida did not express EGFP, whereas blastocysts without a zona pellucida and blastocysts with hatching expressed EGFP (Supplementary Figure S1D, 1E).

**Figure 4:**
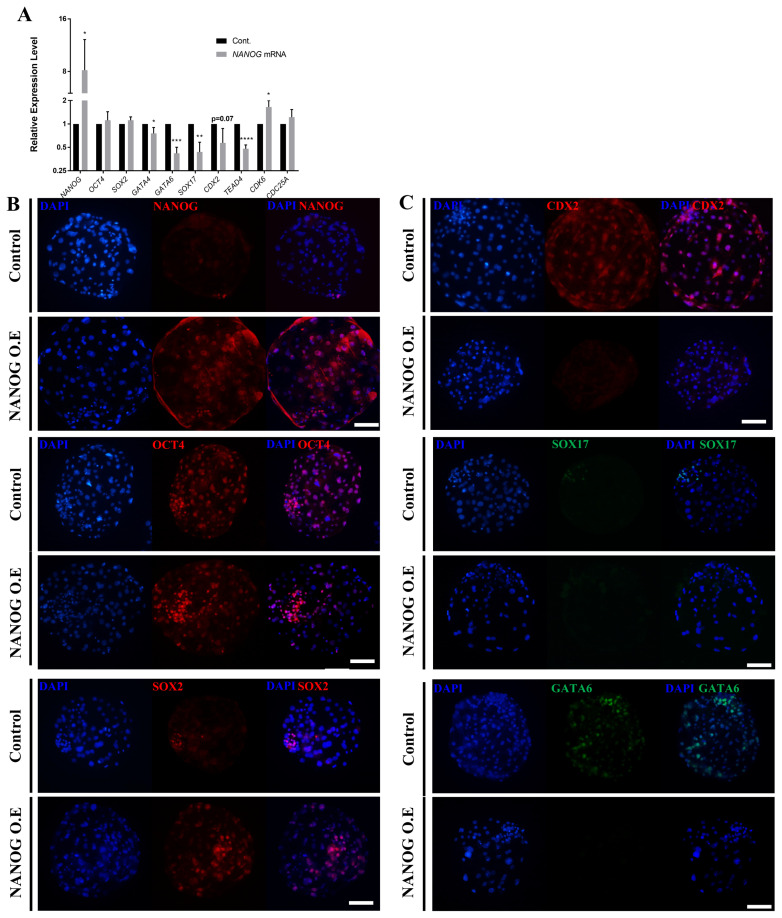
Effects of NANOG overexpression on lineage marker genes in porcine blastocysts. (A) Transcription levels of epiblast, primitive endoderm, and trophectoderm-related genes in control and NANOG mRNA-transfected D7 blastocysts. The sample size was *n* = 30. Each group contained three replicates. Error bars represent the mean standard error of the mean. * Corresponds to significant differences (* p<0.05). (B)–(C) Immunofluorescence analysis for DAPI and NANOG, OCT4, SOX2, and CDX2 (red), as well as SOX17 and GATA6 (green) in control and NANOG mRNA-transfected porcine D7 blastocysts. The size marker corresponds to 100 μm. The sample size was *n* = 10 for each group. NANOG, Nanog homeobox; DAPI, 4′, 6-diamidino-2-phenylindole; OCT4, octamer binding transcription factor-4; SOX2, sex determining region-Y box-2; CDX2, caudal type homeobox 2; GATA6, GATA binding protein 6.

The overexpression assay using *NANOG* mRNA and lipofectamine confirmed that the expression of *NANOG* was higher compared with that of the control group (Figure 4A), whereas that of *SOX2* and *OCT4* was not significantly different from that of the control group. The expression of *GATA4*, *GATA6*, *SOX17*, *CDX2*, and *TEAD4* was significantly decreased in the NANOG overexpression group. On the other hand, the expression of *CDK6* and *CDC25A*, which are related to cell proliferation, was increased. To determine the effect of NANOG on the spatiotemporal expression patterns of other lineage markers, immunofluorescence analysis was performed at the D7 blastocyst stage following NANOG overexpression. First, overexpression was successfully achieved as most of the blastomeres expressed NANOG (Figure 4B). With respect to OCT4 and SOX2, cells with high staining intensity were observed beyond the ICM borderline compared with the control group. CDX2, SOX17, and GATA6 were suppressed in D7 blastocysts by NANOG overexpression (Figure 4C). There was no significant difference in the blastocyst formation rate between the control group and the NANOG overexpression group in the NANOG overexpression assay (Supplementary Table S4). These results indicate that the overexpression of NANOG in porcine embryos increases the expression of pluripotent-related genes and inhibits differentiation into a primitive endoderm and trophectoderm lineage.

## DISCUSSION

### Role of porcine NANOG in lineage specification

In this study, we determined the role of NANOG in epiblast lineage development, maintenance of pluripotency, and the trophectoderm and primitive endoderm lineage in porcine blastocysts. Although NANOG is a core pluripotent transcription factor, detailed studies regarding its role and relationship with other genes during porcine pre-implantation embryogenesis are lacking. Unlike GATA6, we found that the expression of SOX17 was decreased in the NANOG-targeted blastocyst. This is consistent with a study in mouse Nanog-null embryos, in which NANOG activity was required for SOX17, but not GATA6, expression [12]. In addition, the expression of SOX17 and GATA4 was rescued by treatment with Fgf4 in Nanog-mutant embryos. However, in bovine embryos, NANOG knockout embryos expressed SOX17, which indicates species-specific characteristics [21]. Because the expression of Gata4 was decreased in Nanog-null mouse embryos [30], further studies are needed in porcine embryonic primitive endoderm development. Taken together, the expression of the NANOG-related *SOX17* and *GATA6* genes at the early blastocyst stage may be regulated in different ways. Furthermore, GATA6 alone is not sufficient for intact primitive endoderm formation and NANOG plays an essential role in primitive endoderm decision. In addition, NANOG-related primitive endoderm formation also exhibits species-specific characteristics. Therefore, the roles and networks of various genes involved in second lineage specification need to be further studied by species.

NANOG overexpression using lentivirus in the previous Bou’s report [31] is different from the method of this study, so there may be differences in efficiency or results. In this study, overexpression of NANOG did not increase the mRNA expression of SOX2 and OCT4, but immunostaining showed that SOX2 and OCT4 were expressed beyond the ICM boundary, showing similar results in some cases. In the case of GATA6, the expression was decreased in NANOG-overexpressed blastocysts, showing a difference from previous Bou’s report. In mouse blastocysts, NANOG is known to induce epiblasts and GATA6 to primitive endoderms, respectively, but further studies are needed in pigs [32].

The results of this study and previous ones demonstrate that NANOG is associated with the TE markers CDX2 and TEAD4 [31]. In D7 blastocysts overexpressing NANOG, the expression of *CDX2* and *TEAD4* mRNA was decreased and the expression of CDX2 protein was also decreased. In the knockout assay, NANOG was not associated with ICM formation; however, CDX2-positive cells were observed in the ICM (Figure 3A, 3C). Also, in the NANOG overexpression assay, CDX2-poisitive cells disappeared and trophectoderm expression was reduced (Figure 4A, 4C). Previous studies demonstrated that NANOG can regulate the expression of CDX2 in mouse ES cells and plays a subservient role during ICM formation [7]. However, there was no effect on ICM/TE lineage segregation in mouse Nanog-mutant embryos [7,33]. In contrast, porcine blastocysts express OCT4 in the trophectoderm and it is co-expressed with CDX2 [34]. Therefore, NANOG plays a subservient role in suppressing CDX2 expression in the early stage of porcine ICM formation. Taken together, NANOG is expressed later than OCT4 and SOX2 in mammalian embryos but contributes to normal lineage specification.

### Role and expression pattern of OCT4, SOX2, and NANOG on pluripotency in porcine pre-implantation blastocysts

**Figure 5:**
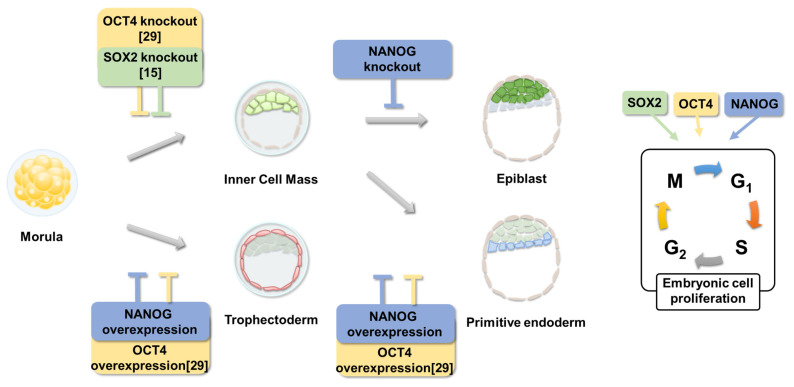
Overview of the role of lineage markers in porcine pre-implantation embryos.

In previous studies, we found that SOX2 plays an essential role in ICM formation [15] and OCT4 also has an essential role in the ICM, trophectoderm, and primitive endoderm development in porcine blastocysts [29]. In addition, SOX2 and OCT4 contribute to cell proliferation in porcine embryos. In this study, we revealed that NANOG plays an essential role in intact lineage specification, pluripotency, and embryonic cell proliferation. To summarize these studies, because blastocysts lacking *OCT4* [29] and *SOX2* [15] cannot form an ICM, these two genes are essential for ICM formation (Figure 5). However, in OCT4-targeted blastocysts, the number of SOX2-positive cells decreased [29], whereas OCT4 was expressed in the trophectoderm of SOX2-targeted blastocysts [15]. This suggests that OCT4 is upstream from SOX2 and OCT4 has a broader role than SOX2, because it is also involved in the development of the trophectoderm. NANOG is not expressed in OCT4- and SOX2-targeted blastocysts, so it is considered downstream of these genes; however, the results showing decreased expression of OCT4 and SOX2 in NANOG-targeted blastocysts suggest that NANOG is essential for epiblast specification and pluripotency. Therefore, these three genes may play a central role in pluripotency maintenance and lineage specification in porcine pre-implantation embryos.

## CONCLUSION

We elucidated a role for NANOG in two lineage specifications during porcine pre-implantation embryogenesis. NANOG maintains embryonic pluripotency, is involved in primitive endoderm and trophoectoderm lineages, and is also implicated in embryonic cell proliferation. Expression of the primitive endoderm marker SOX17 was reduced in NANOG-targeted blastocysts, and the intact primitive endoderm was not formed in NANOG-overexpressing blastocysts. In addition, the trophectoderm did not properly differentiate from NANOG-overexpressing blastocysts. However, more research is needed to compare lineage specifications between species in which certain molecular mechanisms and gene expression patterns differ. Finally, our findings provide insight into the pluripotent network of mammalian embryogenesis and provide experimental models for the study of pre-implantation embryos.

## Figures and Tables

**Table 1:** The number of lineage marker positive cells in NANOG targeted blastocysts

Group	Cell in blastocysts			

	OCT4 positive cells	SOX2 positive cells	SOX17 positive cells	GATA6 positive cells
Cas9 mRNA	122.9±6.9^a^	12.8±1.2^a^	7.2±0.6	67.8±10.7
Cas9 mRNA+sgRNA	33±6.8^b^	0.2±0.1^b^	7.0±0.7	65.0±7.6

NANOG, Nanog homeobox; OCT4, octamer binding transcription factor-4; SOX2, sex determining region-Y box-2; GATA6, GATA binding protein 6.

The number of blastocysts used for each condition was 10.

The number of cells was counted in the late blastocyst.

Data presented as mean±standard error.

a,b Values in a column with different letters are significantly different (p<0.05).

## Data Availability

The datasets used in the current study are available from the corresponding author on reasonable request by email.

## SUPPLEMENTARY MATERIAL

**Supplementary Figure S1.** Experimental design for late overexpression in porcine embryos

**Supplementary Table S1.** Primers used in this study

**Supplementary Table S2.** Oligonucleotide sequences used in quantitative PCR

**Supplementary Table S3.** List of antibodies

**Supplementary Table S4.** Embryo cleavage rates and blastocysts formation rates in NANOG knockout and overexpression assays
