# Supplementary material for: NANOG expression in parthenogenetic porcine blastocysts is required for intact lineage specification and pluripotency
Source: Anim Biosci. 2023 Aug 28;36(12):1905–17. doi: 10.5713/ab.23.0210 (PMC10623019; doi:10.5713/ab.23.0210)
Supplement: Supplementary file 1 [file ab-23-0210-Supplementary-Fig-1.pdf]

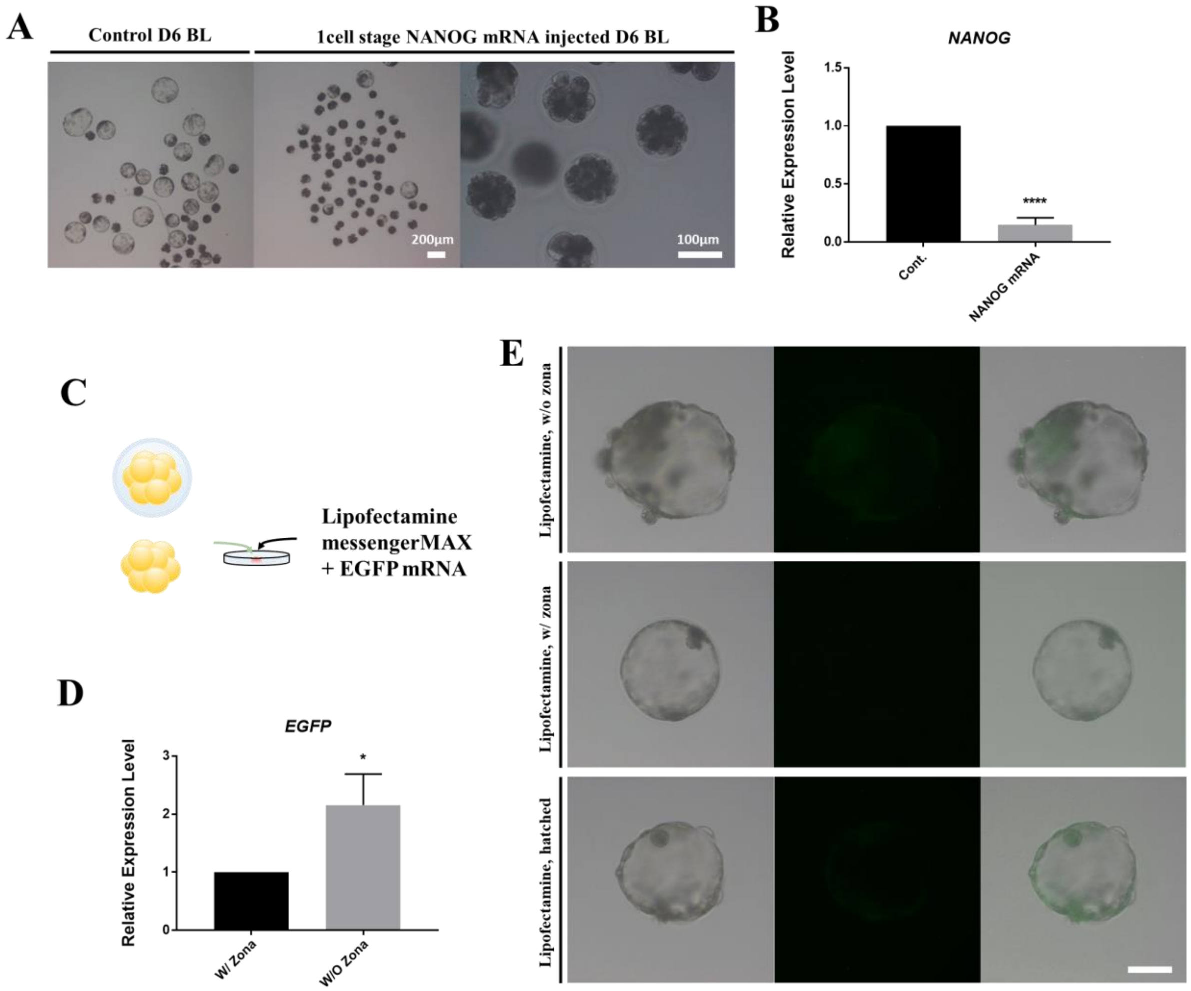

Figure S1. Experimental design for late overexpression in porcine embryos

(A) Control and NANOG mRNA-injected porcine D6 embryos at the one-cell stage.

(B) Transcription levels of control and NANOG mRNA-injected D6 blastocysts. The sample size was n = 30. Each group contained three replicates. Error bars represent the mean SEM. * corresponds to significant differences (***: $P < 0.001$)

(C) Experimental scheme of the overexpression assay using lipofectamine and the EGFP mRNA complex in eight-cell stage porcine embryos.

(D) Transcription levels of EGFP mRNA-transfected D7 blastocysts with and without zona pellucida. The sample size was n = 30. Each group contained three replicates. Error bars represent the mean SEM. * corresponds to significant differences (*: $P < 0.05$).

(E) EGFP mRNA-transfected D7 blastocysts with and without zona pellucida were hatched. Sample size

was $n = 30$.
