# Supplementary material for: NANOG expression in parthenogenetic porcine blastocysts is required for intact lineage specification and pluripotency
Source: Anim Biosci. 2023 Aug 28;36(12):1905–17. doi: 10.5713/ab.23.0210 (PMC10623019; doi:10.5713/ab.23.0210)
Supplement: Supplementary file 2 [file ab-23-0210-Supplementary-Table-1.pdf]

Table S1 Primers used in this study.

Primers	Sequence (5'→3')	Description
pCAG-pNANOG	F: tgaggatccgctagcctgcaGCAGAAGTACCTCAGCCTCC	pCAG-EG(porcine NANOG)FP construction
	R: atcgaattcgtcgacctgcaTTGCTCCAAGACTGGCTGTT	
NANOG gRNA-1	F: CACCGCCAGTACAGAATACCCGGGCTTC	gRNA-1 on-target site
	R: AAACGAAGCCCGGGTATTCTGTACTGGC	
NANOG gRNA-2	F: CACCGAAGCGTTCACCAGGCATCCTTGG	gRNA-2 on-target site
	R: AAACCCAAGGATGCCTGGTGAACGCTTC	
NANOG gRNA-3	F: CACCGTCTGATTACCCCACACGGGCAGG	gRNA-3 on-target site
	R: AAACCCTGCCCGTGTGGGGTAATCAGAC	

Underlined nucleotides refer to gene-specific regions, and lowercase letters indicate overhangs.
