# Supplementary material for: NANOG expression in parthenogenetic porcine blastocysts is required for intact lineage specification and pluripotency
Source: Anim Biosci. 2023 Aug 28;36(12):1905–17. doi: 10.5713/ab.23.0210 (PMC10623019; doi:10.5713/ab.23.0210)
Supplement: Supplementary file 3 [file ab-23-0210-Supplementary-Table-2.pdf]

Table S2 Oligonucleotide sequences used in quantitative PCR.

Primer	Sequence (5'→3')	Tm	Size
OCT4	F: GCTGGAGCCGAACCCCGAGG<br>R: CACCTTCCCAAAGAGAACCCCCAAA	68°C	151
SOX2	F: CGGCGGTGGCAACTCTAC '<br>R: TCGGGACCACACCATGAAAG	64°C	100
NANOG	F: CATCTGCTGAGACCCTCGAC<br>R: GGGTCTGCGAGAACACAGTT -	60°C	195
SOX17	F: GCAAGATGCTGGGCAAGT<br>R: TTGTAGTTGGGGTGGTCCTG	60°C	112
CDX2	F: CAGCGGCGGAACCTGTG<br>R: ACTCGGTATTTGTCTTTCGTCCTG	63°C	92
TEAD4	F: AAGGCCGGCACCATTACCT<br>R: CAGCTCATTCCGACCGTACAT	60°C	231
GATA6	F: CGGCCTCTACAGCAAGATGA<br>R: AGTTGGCACAGGACAATCCA	60°C	98
GATA4	F: GACCACCACCACCACGCT<br>R: AATCCCCTCTTTCCGCATT	60°C	121
ACTB	F: GTGGACATCAGGAAGGACCTCTA<br>R: ATGATCTTGATCTTCATGGTGCT	60°C	131
CDK6	F: CTTTCCACTCCAAATCTCCCCA<br>R: AGAAGCAGGTCTTTGCCTTGT	60°C	76
CDC25A	F: ACCCCAGACTCCATTACCCA<br>F: CAGACGGCTGTACATCTCCC	60°C	200
