# Supplementary material for: NANOG expression in parthenogenetic porcine blastocysts is required for intact lineage specification and pluripotency
Source: Anim Biosci. 2023 Aug 28;36(12):1905–17. doi: 10.5713/ab.23.0210 (PMC10623019; doi:10.5713/ab.23.0210)
Supplement: Supplementary file 4 [file ab-23-0210-Supplementary-Table-3.pdf]

Table S3 List of antibodies.

Primary Antibodies	Target	Host	Company	Catalog Number
	SOX2	Rabbit	Millipore	AB5603
	OCT4	Rabbit	Santa Cruz	sc-9081
	NANOG	Rabbit	Peprotech	500-P236
	SOX17	Goat	R&D systems	AF1924
	GATA6	Goat	R&D systems	AF1700
	CDX2	Rabbit	Abcam	AB7654
Secondary Antibodies	Fluorescent dye	Target/Host	Company	Catalog Number
	Alexa594	Rabbit/Goat	Invitrogen	A-11012
	Alexa594	Rabbit/Donkey	Invitrogen	A-21207
	Alexa488	Rabbit/Chicken	Invitrogen	A-21441
	Alexa488	Goat/Donkey	Invitrogen	A-11055
	Alexa555	Goat/Donkey	Invitrogen	A-21447
	Alexa555	Rabbit/Goat	Invitrogen	A-21428
	Alexa647	Goat/Donkey	Invitrogen	A-32849
