# Supplementary material for: NANOG expression in parthenogenetic porcine blastocysts is required for intact lineage specification and pluripotency
Source: Anim Biosci. 2023 Aug 28;36(12):1905–17. doi: 10.5713/ab.23.0210 (PMC10623019; doi:10.5713/ab.23.0210)
Supplement: Supplementary file 5 [file ab-23-0210-Supplementary-Table-4.pdf]

Table S4 Embryo cleavage rates and blastocysts formation rates in NANOG knockout and overexpression assays.

Group	No. embryos (n=5)	No. cleaved (%)	Blastocyst (%)
**Control (Cas9 mRNA)**	306	220 (72.7 ± 0.02)	91 (30.2 ± 0.01)
**NANOG targeted**	312	211(68.5 ± 0.04)	85 (27.5 ± 0.02)

Group	No. 4 cell stage embryos (n=6)	Blastocyst (%)
**Control (empty)**	310	61 (19.3 ± 0.01)
**NANOG O.E**	327	62 (18.8 ± 0.01)
